# Altered Functional Connectivity Following an Inflammatory White Matter Injury in the Newborn Rat: A High Spatial and Temporal Resolution Intrinsic Optical Imaging Study

**DOI:** 10.3389/fnins.2017.00358

**Published:** 2017-07-04

**Authors:** Edgar Guevara, Wyston C. Pierre, Camille Tessier, Luis Akakpo, Irène Londono, Frédéric Lesage, Gregory A. Lodygensky

**Affiliations:** ^1^Terahertz Science and Technology National Lab, CONACYT-Universidad Autónoma de San Luis Potosí, Coordinación para la Innovación y Aplicación de la Ciencia y la TecnologíaSan Luis Potosí, Mexico; ^2^Sainte-Justine Hospital and Research Center, Department of Pediatrics, Université de MontréalMontreal, QC, Canada; ^3^Montreal Heart Institute, Research CenterMontreal, QC, Canada; ^4^Department of Electrical Engineering, École Polytechnique de MontréalMontreal, QC, Canada; ^5^Department of Pharmacology, Université de MontréalMontreal, QC, Canada; ^6^Department of Neuroscience, Université de MontréalMontreal, QC, Canada

**Keywords:** white matter injury, inflammation, prematurity, resting state functional connectivity, optical imaging of intrinsic signals, support vector machines, artificial neural networks

## Abstract

Very preterm newborns have an increased risk of developing an inflammatory cerebral white matter injury that may lead to severe neuro-cognitive impairment. In this study we performed functional connectivity (fc) analysis using resting-state optical imaging of intrinsic signals (rs-OIS) to assess the impact of inflammation on resting-state networks (RSN) in a pre-clinical model of perinatal inflammatory brain injury. Lipopolysaccharide (LPS) or saline injections were administered in postnatal day (P3) rat pups and optical imaging of intrinsic signals were obtained 3 weeks later. (rs-OIS) fc seed-based analysis including spatial extent were performed. A support vector machine (SVM) was then used to classify rat pups in two categories using fc measures and an artificial neural network (ANN) was implemented to predict lesion size from those same fc measures. A significant decrease in the spatial extent of fc statistical maps was observed in the injured group, across contrasts and seeds (^*^p = 0.0452 for HbO_2_ and ^**^p = 0.0036 for HbR). Both machine learning techniques were applied successfully, yielding 92% accuracy in group classification and a significant correlation *r* = 0.9431 in fractional lesion volume prediction (^**^p = 0.0020). Our results suggest that fc is altered in the injured newborn brain, showing the long-standing effect of inflammation.

## Introduction

Functional connectivity, defined as the coordination of activity across anatomically distinct regions of the brain (Aertsen et al., 1989; Friston et al., 1993), is based on assessment of neuronal activity, either directly, through electrophysiological signals, or indirectly, via hemodynamics or metabolites fluctuations.

Functional MRI (fMRI), arguably the most commonly used technique to assess brain activation, relies on the measurement of blood-oxygen-level-dependant (BOLD) signals. It reflects changes of blood volume and deoxyhemoglobin (HbR) concentrations (Buxton, 2013). fMRI was first described by Ogawa in 1990 (Ogawa and Lee, 1990; Ogawa et al., 1990) and has since revolutionized human neuroscience by allowing the identification of brain areas responsible for diverse cognitive task or processing various stimuli (Belliveau et al., 1991; Raichle, 2009). Resting-state fMRI (rs-fMRI) introduced a few years later (Biswal et al., 1995) corresponds to the measurement of low frequency fluctuations (0.009 < *f* < 0.1 Hz) in the BOLD signal in the absence of a task or stimulus. It has also greatly contributed to the neuroscience field by revealing the existence of multiple spatially distributed large-scale networks in the brain, the resting-state networks (RSN). Lewis and al. have shown that a possible function of RSN could be related to the consolidation of previous experience (Lewis et al., 2009). According to another hypothesis, the networks functioning during active processing are maintained during rest (and therefore become the RSN) in order to allow rapid activation when needed for active processing (Smith et al., 2009). However, the true function of the RSN remains unknown.

The application of fMRI or rs-fMRI in animal models has been scarce and mostly limited to rats and monkeys, because smaller models such as mice or neonatal rats require a very high intensity magnetic field to obtain sufficient signal-to-noise ratio and spatial resolution to assess their small brains (Benveniste and Blackband, 2002; Jonckers et al., 2011). Given the tremendous number of already well-characterized models that can't be readily studied because of this limitation, interest for the other functional neuroimaging techniques, alone or in combination with fMRI, has increased over the past years (Cang et al., 2005; He and Liu, 2008; Ye et al., 2016). Optical imaging of intrinsic signals (OIS) was shown to be a potent alternative to fc-MRI, also by measuring changes in blood oxygenation (Grinvald et al., 1986; Frostig et al., 1990; Ts'o et al., 1990). In this technique, two wavelengths based on the absorption spectra of oxyhemoglobin (HbO_2_) and deoxyhemoglobin (HbR), are shone upon exposed nervous tissue and fluctuations in reflected light intensity are recorded. Those fluctuations represent changes in blood oxygenation/volume and, therefore, can be used to infer changes in neuronal activity. Advantages of OIS over (rs-)fMRI include a much higher spatial resolution (in the order of tens of micrometers for OIS vs. 200–400 μm for fMRI) and temporal resolution (30 Hz for OIS vs. 0.5 s for high-resolution fMRI sequences; Pouratian and Toga, 2002; Jonckers et al., 2015; Pan et al., 2015), providing an interesting tool to assess functional connectivity in small animals models. Unlike fMRI, OIS is not prone to artifacts related to mechanical vibrations and spurious responses arisen from loud acoustic stimuli. Moreover, OIS also uses non-ionizing radiation and is much more cost-effective than fMRI. However, one major limitation of OIS is its inability to assess subcortical structures (Hillman, 2007). The resting state data, acquired with this technique, is hence 2 dimensional. Nevertheless, most of the RSN have some, if not all, of their brain regions in the cortex (Rosazza and Minati, 2011), making OIS a suitable method to study RSNs in small models, like rodents (Li et al., 2012; Guevara et al., 2013c).

The study of alterations in RSN via rs-fMRI has improved our understanding of many neurological and psychiatric diseases such as epilepsy (Wang Z. et al., 2011; Mankinen et al., 2012; Tracy and Doucet, 2015), depression (Mulders et al., 2015), autism (Farrant and Uddin, 2016; Mevel and Fransson, 2016), schizophrenia (Sheffield and Barch, 2016), and dementia (Greicius et al., 2004; Gili et al., 2011; Peraza et al., 2015). The impact of prematurity on neurocognitive development has also been, more recently, studied through fMRI and rs-fMRI. Studies have identified resting state network maturation in the growing brain with evidence of networks as early as 30 weeks of gestational age with a fast maturation leading to adult-like network at term equivalent age (Doria et al., 2010; Smyser et al., 2010; Lee et al., 2013; van den Heuvel et al., 2015; He and Parikh, 2016). The consequences of prematurity itself was revealed by showing a decreased functional connectivity of RSN in (very preterm) VPT-born patients (Smyser et al., 2010, 2013; Ye et al., 2016), best seen using correlation and covariance matrix analyses demonstrated by Smyser et al. (2016b). In other words, the topography of RSN in VPT-born children seems preserved, but quantitative parameters, such as the synchronicity between networks (White et al., 2014; Ye et al., 2016), the amplitude of BOLD signal fluctuations (Smyser et al., 2013, 2016b) or the number of voxels in a RSN (Smyser et al., 2010), appeared to be decreased with a more pronounced reduction in higher order RSN (Default mode, executive control, and frontoparietal networks; Smyser et al., 2016b). Most studies reported that primary RSN (somatosensory, motor, and visual) are almost fully formed (i.e., adult-like) before week 30 of PMA while higher order networks mature during the last trimester (Doria et al., 2010).

The exploration of RSN in VPT children is still in its very first steps. Studies have investigated connectivity in the first 4 years of life of children born preterm (Lee et al., 2013) and in adults and older children born preterm (White et al., 2014; Ye et al., 2016). In spite of the useful information obtained through cohort studies, preclinical models are crucial to test neuroprotective treatment. However, as stated earlier, the data on rodents or other small models remain scarce because of the intrinsic limitations of fMRI. In this study, we characterized using rs-OIS, resting state networks in a preclinical model of diffuse white matter injury that consists in the intra-cerebral injection of lipopolysaccharide (LPS) (Cai et al., 2003; Pang et al., 2003; Lodygensky et al., 2010, 2014; Guevara et al., 2013a). LPS is known to mimic every hallmark of inflammatory white matter injury, in the immature brain such as seen in preterm infants following necrotizing enterocolitis or born in the context of severe chorioamnionitis e.g., hippocampal volume impairment (Wang et al., 2013), ventricle dilation, apoptosis, pre-olygodendrocite cell necrosis, white matter rarefaction, hypomyelination, microglial reaction (Cai et al., 2003; Pang et al., 2003) as well as neurobehavioral deficits (Fan et al., 2005).

## Methods

### Animal model

All procedures were sanctioned by the Institutional Committee for Animal Care in Research of the CHU Sainte-Justine and Montreal Heart Institute Research Centers, and conducted under isoflurane anesthesia to minimize pain and distress, following the recommendations of the Canadian Council on Animal Care.

A total of 15 Sprague-Dawley pups coming from two litters (7.96 ± 0.45 g weight, Charles River, Senneville, Qc, Canada) were randomized in two different groups: LPS (*n* = 6) which were injected at postnatal day 3 (P3, equivalent to human gestational age of about 24–28 weeks (Sizonenko et al., 2003; Lodygensky et al., 2014) with a solution of lipopolysaccharide diluted in saline (1 mg/kg in 0.5 μL, *E. Coli*, serotype 055:B5, Sigma St Louis, MO) and a sham control group NaCl (*n* = 9), injected with saline alone. Injection site was the left corpus callosum at a level equivalent to P-7, c9 (Lodygensky et al., 2014). All injections were performed with an ultrasound-guided micro injector (Micro4 from World Precision Instruments) at a rate of 0.1 μL/min. From these rat pups 2 animals were rejected, one from each group, due to a technical error during data acquisition that was only identified later on; so the final population is LPS (*n* = 5; 1 female, 4 males) and NaCl (*n* = 8; 2 females, 6 males).

### Resting-state optical imaging of intrinsic signals (rs-OIS)

#### Animal preparation

At postnatal day 24 or 25 (P24 or P25, equivalent to human pre-puberty (Chen et al., 2016) the rat pups were fixed onto a small animal physiological monitoring station (Small Animal Monitoring System 75-501 Harvard Apparatus, Holliston, MA) which allowed the restriction of head motion and continuous recording of respiration, heart rate, and closed-loop thermoregulation at 37 ± 0.5°C.

In addition to urethane (2 g/kg), a local anesthetic (Xylocaine 0.2%) was injected and scalp was carefully removed to expose the skull covering the cortex. Artificial cerebrospinal fluid (Bélanger et al., 2016) was poured over the skull to prevent drying and minimize specular reflections due to skull bone. Urethane was chosen over isoflurane due to concerns of its negative impact on resting state activity (Wang K. et al., 2011). Ideally resting state activity should be performed awake but sedation was required due to the surgery. Urethane was chosen as an alternative as it has been used and recommended in fMRI studies (Huttunen et al., 2008; Paasonen et al., 2016) and in previous resting state studies (Wilson et al., 2011; Kozberg et al., 2013).

#### Optical imaging

Imaging was carried out according to previous rs-OIS studies in rodents (Guevara et al., 2013b,c; Bélanger et al., 2016). Briefly, multi-spectral rs-OIS (λ = 525, 590, 630 nm) was performed under anesthesia. Seven minutes of resting state activity were recorded. Time multiplexed illumination (3.5W LED, LZ4-00MA00, Led Engin) yielded a full-frame sampling frequency of 5 Hz. Furthermore, illumination intensity was adjusted to avoid under or over saturated spots of the brain with an exposure time of 30 ms. A charge-coupled device (CCD) camera (MV-D1024E-160-cl, PhotonFocus) with a 12-bit ADC and 1,024 × 1,024 pixels in the image area acquired the images through a macro lens (EF-S 60 mm f/2.8, Macro USM, Canon) over a field-of-view of (14.7 mm)^2^. Using an aperture of 2.8, a depth of field of 1.2 mm was obtained. Image acquisition via a frame grabber (Neon-CLB, Bitflow) was controlled by a custom-made graphical interface developed in MATLAB (The MathWorks, Natick, MA).

#### Seed-based functional connectivity (fc) analysis of rs-OIS

Using the incident light *I*_0_ and the reflected light *I* intensities, multispectral optical density ($\Delta OD=\log\left(\frac{I_{0}}{I}\right)$) images were converted to HbO_2_ and HbR measures *C*_*i*_(*t*) using the modified Beer–Lambert law and a Moore–Penrose pseudoinverse, according to Delpy et al. (1988):

(1) $$\Delta OD\left(\lambda ,\;t\right)=\sum_{i}\in_{i}\left(\lambda \right)C_{i}\left(t\right)D\left(\lambda \right)$$

Where the differential path length factor *D*(λ) values were obtained from the literature (Kohl et al., 2000; Dunn et al., 2005) and hemoglobin extinction coefficients ϵ_*i*_(λ) were obtained from Prahl (1999). OD values were corrected for the wavelength-dependent response of the CCD sensor and convolved with the LEDs profile (Brieu et al., 2010).

In order to minimize the influence of physiological artifacts on the resting state signal, a general linear model (GLM) (Friston et al., 2006) was used with multiple physiological regressors *X*(*t*): heart rate, respiratory signal, ECG, respiration rate, and the average signal of all those pixels identified as belonging to the cortex:

(2) $$\beta_{\text{X}}=\text{X}{\left(t\right)}^{+}{\text{C}}_{\text{i}}\left(t\right)$$

Then the weights β_X_ are used to obtain the residual of the regression ${{\text{C}}_{\text{i}}}^{\prime }\left(t\right)$, that is then used for the fc analysis:

(3) $${\text{C}}_{\text{i}}^{\prime }\left(t\right)={\text{C}}_{\text{i}}\left(t\right)-\text{X}\left(t\right)\;\beta_{\text{X}}$$

HbO_2_ and HbR measures, i.e., ${{\text{C}}_{\text{i}}}^{\prime }\left(t\right)$ were spatially smoothed using an 11 × 11 pixels (~150 × 150 μm) gaussian kernel with a 3 pixels (43 μm) standard deviation. These chromophore signals were then temporally filtered between 0.009 and 0.08 Hz, using a fourth-order Butterworth filter with zero-phase shift, according to previous fc studies (Guevara et al., 2013c). Images were aligned to a reference atlas through a projective registration using Matlab and anatomical landmarks as the control points for such registration.

Regions of interest, also called seeds in the context of fc, were obtained from previous studies in the rodent brain that have identified a series of cortical regions that exhibit alterations in fc in several conditions, such as ischemic stroke (Bauer et al., 2014), epilepsy (Guevara et al., 2013b), arterial stiffness (Guevara et al., 2013c), cortical spreading depression (Li et al., 2012), and amyloid-β deposition (Bero et al., 2012).

All the seeds were placed using atlas coordinates, using data shown by Jung et al. (2013) as shown in **Figure 5A**. Time series of every seed were computed as the average value of 17 pixels (~0.21 mm) around the seed locus. The Pearson correlation values *r* between each seed time trace were used as the metric for seed-to-seed analysis, converted to Fisher *Z*-values using $Z\left(r\right)=\frac{1}{2}\ln\;\left[\frac{1+r}{1-r}\right]$ before doing group level comparisons. Statistical significance was determined by one-sample Wilcoxon rank sum test and *p*-values were false discovery rate (FDR) adjusted (Benjamini and Hochberg, 1995). Effect size was computed with Hedges' g (Hedges and Olkin, 1985). Group-mean connectivity matrices were computed from average seed-to-seed correlation values for both contrasts (HbO_2_ and HbR). Since this model follows a unilateral modification, interhemispheric connectivity values are expected to change, therefore seed-based homotopic connectivity was also investigated as a metric to corroborate those changes, as previous studies (Bero et al., 2012; Guevara et al., 2013a). Seed-to-pixel analysis was carried out by computing the functional correlation between each seed time trace and the time course of the pixels marked as belonging to the brain cortex. Fisher Z transformation was applied to single subject correlation maps, then normalization by subtraction of the mean and division by the standard deviation was performed (Greene et al., 2015). For each normalized seed-based fc map, two-tailed, one sample *t-*test was implemented to characterize the connectivity patterns of each group, assessing its strength relative to zero (Zhan et al., 2014). In order to correct for false positives, a height-threshold of *p* < 0.05, FDR-corrected, was implemented (Genovese et al., 2002). Furthermore, clusters with fewer pixels than 5% of the total number of height-threshold surviving pixels were removed (Warren et al., 2009). In this work we define spatial extent as the ratio of the number of pixels that survived the threshold (FDR-corrected *p* < 0.05 in height and <5% in extent) to the number of pixels marked as belonging to the brain cortex in our anatomical atlas. Fisher's exact mid-P method was implemented to find out if gender was a confounding factor (Thorvaldsen et al., 2010).

#### Machine learning

Machine learning plays an ever increasingly important role in the neuroimaging field, especially in computer assisted diagnosis (Suzuki et al., 2012). Machine learning techniques may be valuable tools in recognizing connectivity patterns that arise from an inflammatory injury. Therefore, in this paper we apply two different machine learning techniques to assess its value in the context of white matter injury.

##### Support vector machine

A support vector machine (SVM) classifier was used to assign labels automatically to rat pups in a blind evaluation, allowing us to explore the research question: Does blind segmentation of subjects using SVM reflect injury status?

Seed-to-seed fc values were chosen as the set of features (56, comprised of both contrasts HbO_2_ and HbR) used as inputs to the SVM classifier depicted in **Figure 5B**, which separated the data into two groups: NaCl and LPS. A radial basis function kernel was chosen and sequential minimal optimization was the method used to find the separating hyperplane. Both the box constraint parameter and the kernel sigma were optimized through a grid-search to obtain better accuracy (Gaspar et al., 2012). The performance of the classifier was determined in terms of sensitivity (Se, the proportion of LPS pups identified as such), specificity (Sp, the proportion of control animals correctly identified), positive predictive value (PPV, the proportion of LPS-labeled pups that are actual lesioned animals), negative predictive value (NPV, the ratio of pups labeled as NaCl that actually belong to the control group), and accuracy (Acc, the proportion of correct labels), which were computed by averaging the results of ten 10-fold cross-validation runs. In each one of the runs, the data was split into 10 approximately equal partitions, and each in turn was used for training while the remainder is used for validation and testing, i.e., the samples were randomly divided as follows: 70% for training (9–11 samples), 15% for validation (1, 2 samples), and 15% (1, 2 remaining samples) as a completely independent testing, for each run, until all samples were independently tested. A choice of *k* = 10 was chosen, as proposed by Borra et al. (Borra and Di Ciaccio, 2010), the number of folds was limited by our sample size.

##### Artificial neural network

An artificial neural network (ANN) was designed to answer the following research question: do fc patterns correlate with lesion size? A set of 56 features comprised of the seed-level connectivity matrix of HbO_2_ and HbR measures was the input to the ANN as shown on **Figure 5D**, i.e., the same set of features used in the SVM classifier described above. A two-layer feed-forward ANN with 20 sigmoid hidden neurons and one linear output neuron maps the fc measures to the lesion size. The number of neurons in the hidden layer was chosen according to the method proposed by Huang (2003). The network was trained using Bayesian regularization, which is more robust to small data sets, as in our case (Burden and Winkler, 2008). Samples from both groups were randomly divided as follows: 70% for training (7 samples), 15% for validation (1 sample), and 15% as a completely independent testing (1 sample). The ANN was trained to a maximum of 1,000 epochs and the mean square error goal was assigned to 0.00001. Accuracy of the ANN was evaluated using correlation coefficient (r) and root-mean square error (RMSEP).

### Histology

Rat pups were perfused through the heart with phosphate-buffered saline solution (PBS) followed by 4% paraformaldehyde. LPS and sham-injected brains were removed and immersed in paraformaldehyde at 4°C for 24 h and then transferred to 30% sucrose solution for 2 days prior to cryo-sectioning.

Measurements were performed on coronal rat brain sections, 300 μm apart, stained with cresyl violet and scanned with Axioscan Z1 (Carl Zeiss Inc, ON, Canada). Each coronal section (50 μm thick) was thresholded, binarized, and holes were filled in order to delineate the ventricular region using Fiji (Schindelin et al., 2012). Pixels were counted and converted to linear units (228 pixels/mm), the area was multiplied by the slice thickness and all slices were added to find the ventricular volume (Wang et al., 1997). Fractional measurements were performed in order to account for variability in brain size and deformation of histological slices. Due to the fact that ventricles were barely discernable in NaCl pups, only four of them were analyzed.

## Results

The optical intrinsic signal obtained from young rat allowed the characterization of resting state homeostasis in normal and inflammatory conditions. Three complementary approaches were used: (i) Seed-based functional connectivity analysis of rs-OIS; (ii) Support vector machine; (iii) Artificial Neural Network.

### Seed-based functional connectivity analysis of rs-OIS

**Figure 1:**
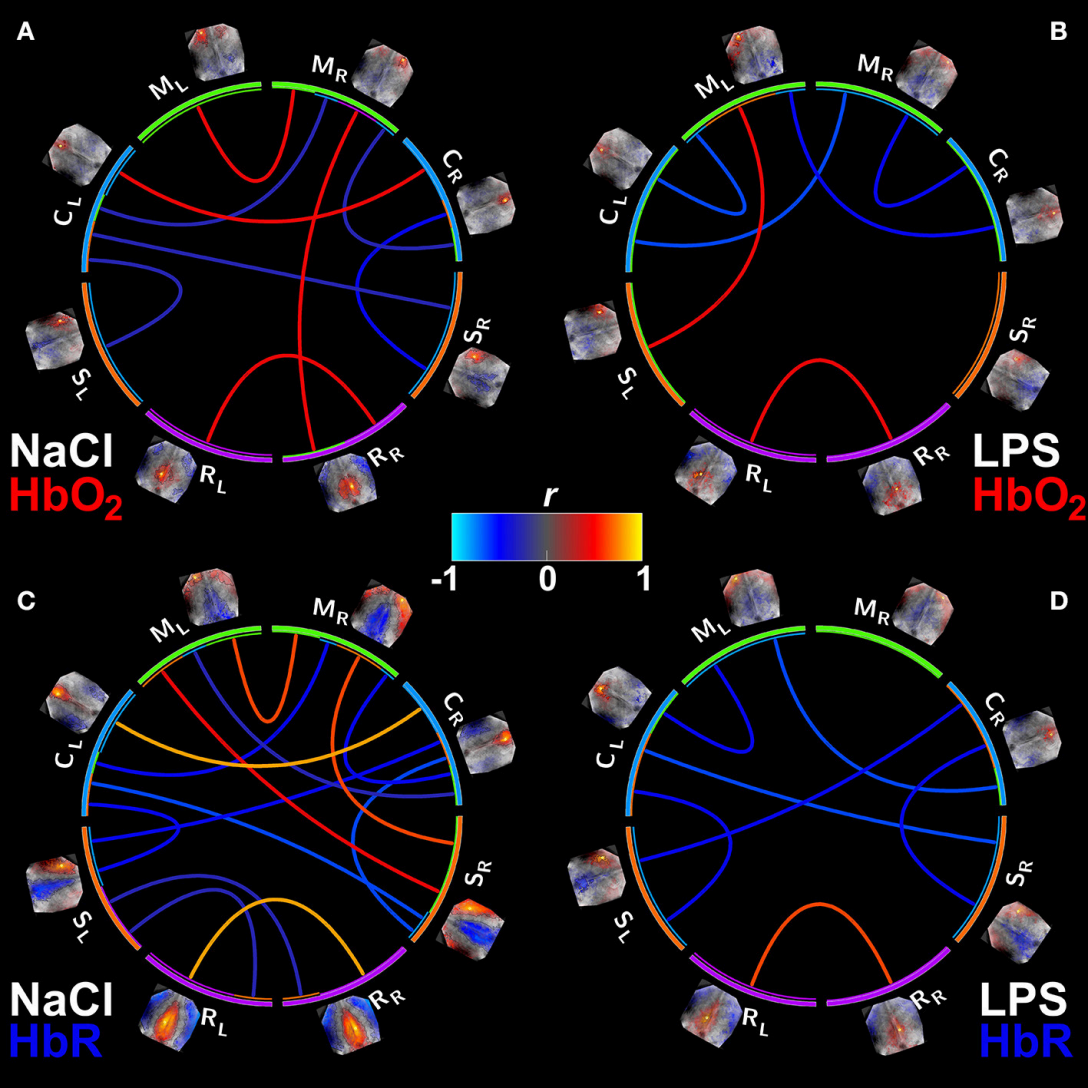
Group mean connectivity matrices visualized with Circos (Krzywinski et al., 2009). **(A,B)** Show the connectivity matrices for HbO_2_ contrast from the NaCl and LPS groups, respectively. **(C,D)** Same as **(A,B)** but for HbR. Supra-threshold edges are shown (FDR-corrected *p* < 0.05). M, motor cortex; C, cingulate cortex; S, somatosensory cortex; R, retrosplenial cortex. Subscripts L and R indicate hemisphere side. Note the significant reduction in connectivity in animals exposed to inflammation with a larger effect on HbR contrast.

Connectivity matrices for both groups and both contrasts (HbO_2_ and HbR) were evaluated (Figure 1). When identifying seed based functional connectivity analysis of rs-OIS in sham animals, connectivity matrices were better identified in HbR contrast (Figure 1).

The association between gender and fc measures was not statistically significant (*p* = 0.7552). As displayed on the connectivity matrices, our results show that homotopic fc values in the motor cortex and cingulate region are impaired in the injured group; for HbR contrast homotopic connectivity in retrosplenial region is decreased as well. Positive connections are less numerous in the LPS group, and this diminution is more remarkable in HbR connectivity matrices, as shown in Figure 1D. This decrease of fc was also present in the anti-correlated edges. Yet, there were no statistical differences in seed-to-seed connectivity between the groups, after FDR correction (data not shown).

### Spatial connectivity extent

**Figure 2:**
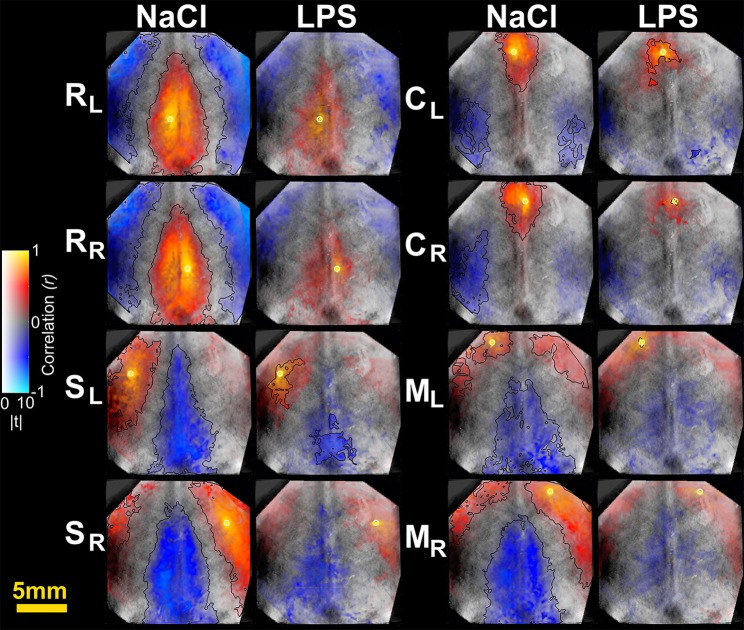
Seed-based correlation maps for HbR contrast. T-statistics are encoded as transparency, while the correlation values are encoded as hue values (Allen et al., 2012). A height-threshold of *p* < 0.05, FDR-corrected, was implemented. Clusters with fewer pixels than 5% of the total number of suprathreshold pixels were removed (Warren et al., 2009). A black contour is shown for FDR-corrected *p* < 0.05 and spatial extent threshold >5%. Note the decrease of significantly correlated pixels in the LPS group. M, motor cortex; C, cingulate cortex; S, somatosensory cortex; R, retrosplenial cortex. Subscripts L and R indicate hemisphere.

**Figure 3:**
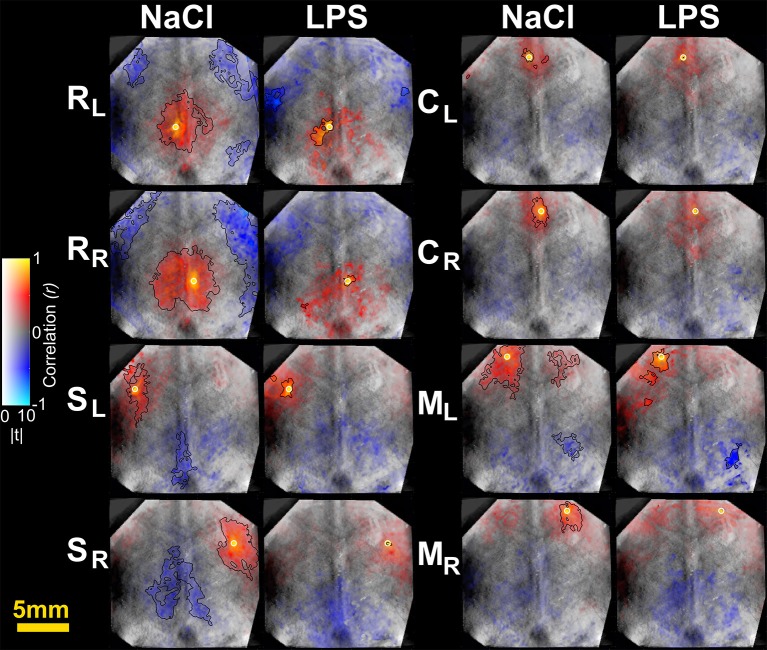
Seed-based correlation maps for HbO_2_ contrast. T-statistics are encoded as transparency, while the correlation values are encoded as hue values (Allen et al., 2012). A height-threshold of *p* < 0.05, FDR-corrected, was implemented. Clusters with fewer pixels than 5% of the total number of suprathreshold pixels were removed (Warren et al., 2009). A black contour is shown for FDR-corrected *p* < 0.05 and spatial extent threshold >5%. Note the decrease of significantly correlated pixels in the LPS group. M, motor cortex; C, cingulate cortex; S, somatosensory cortex; R, retrosplenial cortex. Subscripts L and R indicate hemisphere.

**Figure 4:**
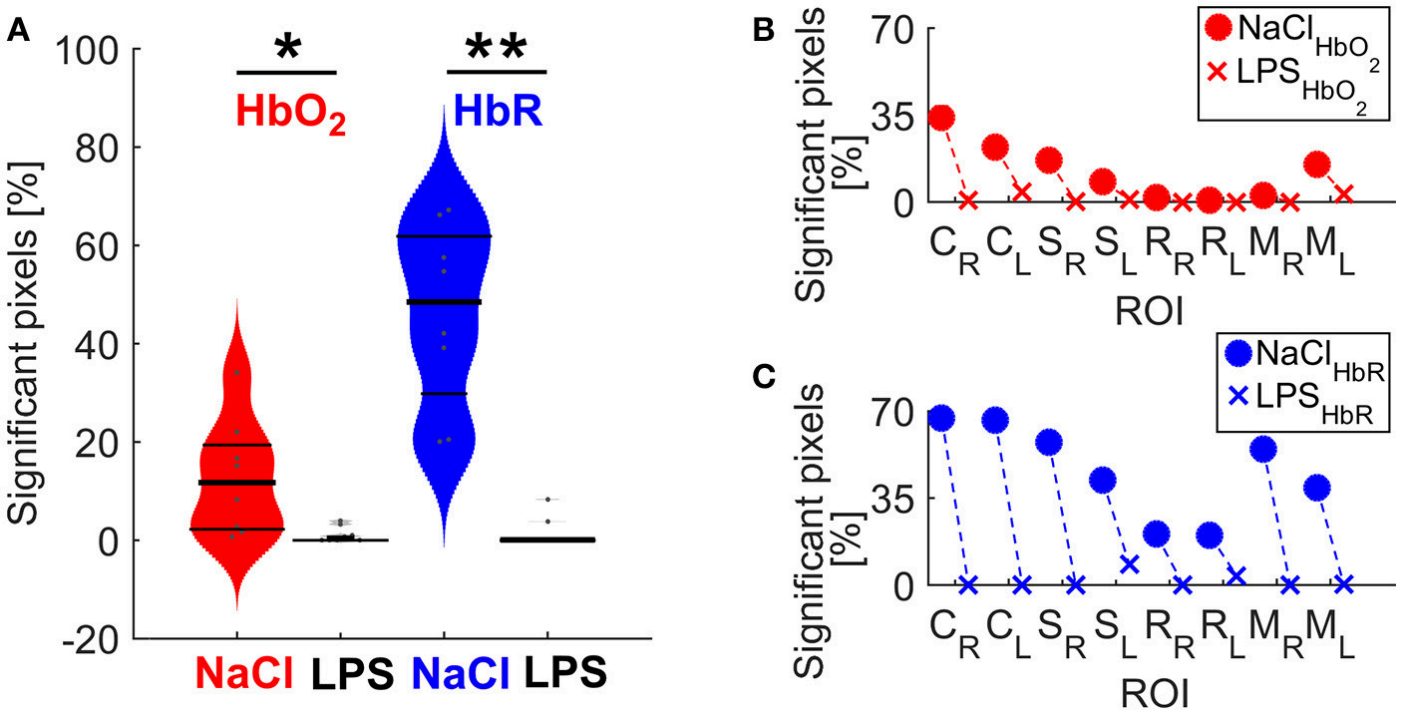
**(A)** Spatial extent differences between NaCl and LPS groups. The shape of each distribution is plotted, overlaid with data points and lines denoting 25, 50, and 75 percentiles. ^*^P = 0.0452 and Hedges' *g* = 0.9338 for HbO_2_; ^**^p = 0.0036 and Hedges' *g* = 1.0861 for HbR. **(B)** Spatial extent by individual networks for HbO_2_ contrast **(C)** Spatial extent by individual networks for HbR contrast. M, motor cortex; C, cingulate cortex; S, somatosensory cortex; R, retrosplenial cortex. Subscripts L and R indicate hemisphere.

To further characterize the impact of cerebral inflammation in the developing brain we studied the spatial extent of networks. We generated group-averaged fc maps for HbR and HbO_2_ contrasts with seeds placed in the motor cortex, the cingulate cortex, the somatosensory cortex and the retrosplenial cortex (Figures 2, 3). Inflammation caused a significant decrease of spatial extent across cortical regions, when compared to the NaCl group (LPS = 1.5 ± 1% vs. NaCl = 45.9 ± 0.6%, ^**^p = 0.0036, Hedges' *g* = 1.0861, as shown in Figure 4A). A similar reduction is observed in HbO_2_ fc maps displayed in Figure 3 (LPS = 1.1 ± 0.6% vs. NaCl = 12.7 ± 4.1%, ^*^p = 0.0452, Hedges' *g* = 0.9338, as shown in Figure 4A). A large effect size was detected in both contrasts. Moreover, this decrease is consistent across seed locations and intrinsic contrasts, as shown in Figures 4B,C. These results indicate decreased functional connectivity following white matter injury across different regions and contrasts.

### Machine learning analysis of rs-OIS using support vector machine to identify injury

**Figure 5:**
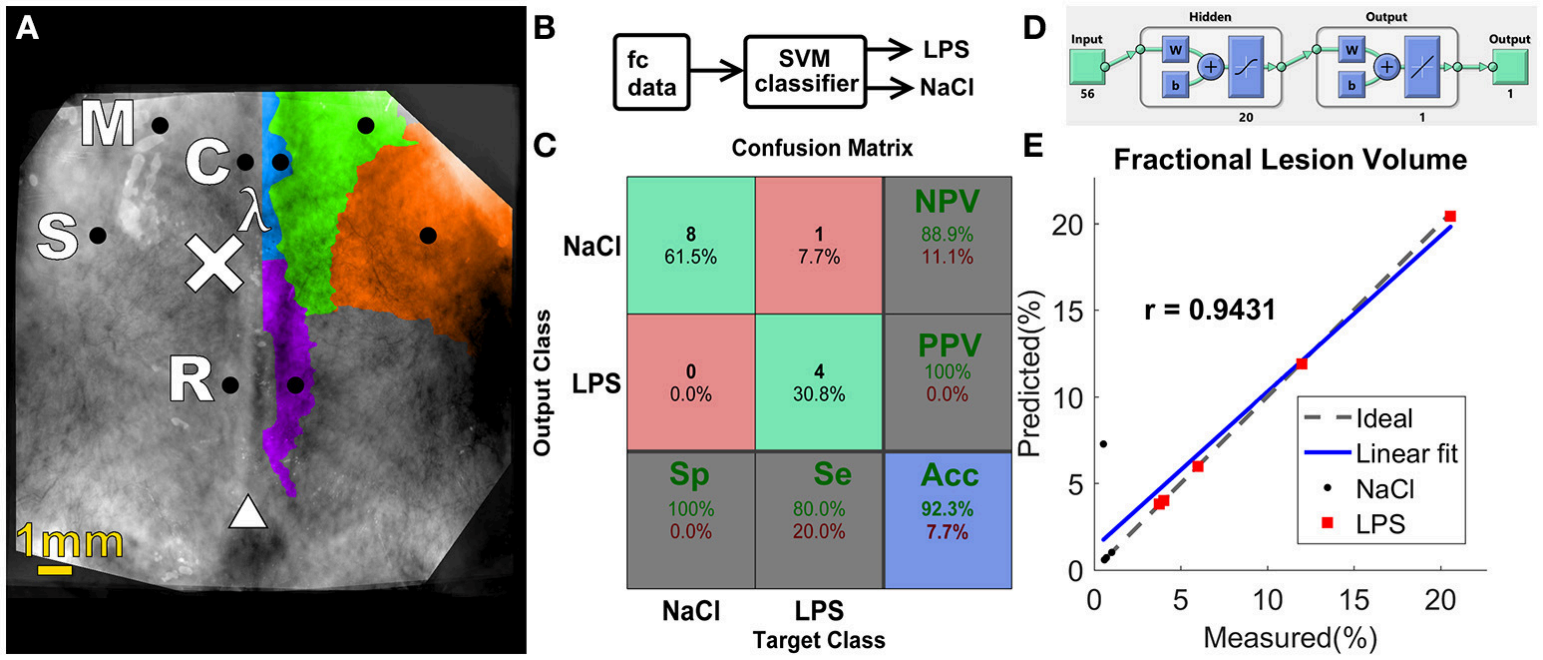
**(A)** Functional regions on the rat cortex, seed placement and size, manually constructed from data shown by Jung et al. (2013). Black circles indicate seed position and size. Injection site is denoted by the “×” symbol, lambda denoted by λ and bregma indicated by triangle symbol. M, motor; C, cingulate; S, somatosensory; R, retrosplenial. **(B)** Block diagram of the proposed classifier **(C)** Confusion matrix for the SVM classifier. **(D)** Graphical diagram of the ANN. **(E)** Regression plot for ANN predicted ventricular size vs. measured ventricular size.

Separating the data into two groups: sham animals and injured ones, we built a confusion matrix for the SVM binary classifier using the set of HbO_2_+ HbR features (Figure 5B), following the cross-validation procedure mentioned in the Methods Section. Only one subject from LPS group was incorrectly classified as belonging to NaCl group. This resulted in a 92.3% accuracy (Figure 5C). Other kernels, such as a linear kernel did not yield higher accuracy and therefore are not presented in this work.

### Machine learning analysis of rs-OIS using an artificial neural network to quantify injury

The implementation of an ANN model (Figure 5D) enabled the prediction of fractional lesion volume based on fc-OIS measures. In similar fashion to SVM classifier described above, the connectivity matrix comprised of both HbO_2_ and HbR contrast, totaling 56 seed-to-seed connectivity values, was used as input to our ANN. Using Bayesian regularization as our training algorithm, we found a good agreement between the results predicted by this model and the degree of ventricular dilatation, (regression coefficient *r* = 0.9431, *p* = 0.0020, and a RMSEP = 2.25%; Figure 5E). Other back-propagation algorithms such as Levenberg–Marquardt training failed to predict injury accurately (results not shown). The number of neurons in the hidden layer was chosen as a compromise in prediction error and computation time. An increase in the number of neurons did not result in better performance, and architectures with fewer neurons did not yield accuracy >90%, thus 20 neurons was a justifiable choice.

## Discussion

Optical intrinsic signal represents a major advantage over fcMRI due to a very high temporal and spatial resolution. It is straightforward to setup compared to MRI, with neither associated mechanical vibrations nor loud sounds, thus avoiding vibration and acoustic noise-related artifacts; while providing better access to the animal, thus facilitating its manipulation. It is obviously restricted to 2D mapping and not a technique of choice for deep brain structures. This is an important limitation when performing regression on rs-OIS data; there is the possibility that global signal regression leads to spurious negative correlations (Murphy et al., 2009; Saad et al., 2012), although some works suggest a physiological basis for anticorrelated networks (Fox et al., 2009). Unfortunately, a viable alternate option is not provided by the OIS technique. Further work is needed on this issue.

As shown in adult mice (Guevara et al., 2013c) we found also that HbR contrast appears to be more sensitive to uncover resting state networks in the young rat brain (Figure 1). Interestingly HbR has been shown to closely match the BOLD contrast in fMRI experiments (Huppert et al., 2006).

When compared to adult mice (Guevara et al., 2013c), rs-OIS in young rats detected similar functional correlation maps, across different seeds, showing high degree of inter-hemispheric symmetry in control animals.

In the developing rat brain, an inflammatory brain injury was found to cause significant long standing disruption of resting state activity using rs-OIS with a reduction of connectivity in several networks particularly on motor and cingulate networks. The clear impact of inflammation on these networks is not unexpected considering the site of LPS injection. Nevertheless, the identification of persisting deficits is remarkable as these results are in agreement with preterm infants with substantial white matter injury, with a significant reduction on motor networks and in the vicinity of clear injury (Smyser et al., 2013).

When studying averaged seed-based correlation maps, inflammation was shown to cause a significant decrease of spatial extent across cortical regions, that was highly significant on HbR maps (Figures 2–4). This difference in HbR with respect to HbO_2_ may be explained by the higher contribution of HbR to absorption at 590 and 630 nm wavelengths. As mentioned in the introduction similar reduction of correlated voxels where found in preterm infants when compared to term infants imaged at term equivalent age (Smyser et al., 2010). Translating this approach on pre-clinical fcMRI in young developing rat or mice brains might prove difficult as partial volume effect might dampen differences between groups especially in small correlated regions such as in the cingulate cortex, where fc-OIS was able to find a difference of ~0.6 mm^2^. This small, albeit significant difference might be challenging to unravel using fcMRI in rodents, where voxel size is usually between 0.5 mm^3^ (Liang et al., 2012) and 1 mm^3^ (Harris et al., 2015).

The use of a SVM allowed us despite the limited sample size, to efficiently classify injured from intact developing brains with accuracy above 90%. In contrast to seed based analysis it provides a categorical result with no indication regarding the type of changes. The advantages and limitations still need to be properly addressed especially if used to determine therapeutic efficiency. Nevertheless, it is a first step in detecting injury through optical imaging, since SVM was shown to identify injury with high accuracy, using rs-OIS data. Similarly, Vergara et al. have used SVM with a leave-one-out cross validation to classify mild traumatic brain injury based on fcMRI with an accuracy of 84.1 % that was superior to DTI analysis (Vergara et al., 2017). This approach was also successfully applied in a cohort of preterm infants also using fcMRI with an 84% accuracy and a specificity of 78% (Smyser et al., 2016a). The range of accuracy probably depends on the severity of injury. It is noteworthy that this approach remains extremely efficient in the setting of mild traumatic brain injury and in a cohort of healthy preterm infants at term with no overt brain injury. The increased temporal resolution of rs-OIS over fcMRI may increase the quality of resting state assessment. It is possible that in young rodent, rs-OIS might reveal itself superior in comparative studies with fcMRI.

The use of an ANN allowed us to assess the degree of injury with a highly significant correlation with the degree of ventricular dilation. Machine learning approaches have proven useful in neonatal populations, e.g., using SVM to predict gestational age (Smyser et al., 2016a), ANN to assess mortality risk (Zernikow et al., 1998) or heuristic methods to detect seizures (Ansari et al., 2016). One limitation of ANN, and machine learning in general, is the fact that the only observed states are the inputs and the outputs, making interpretation of their inner functions exceedingly difficult (Castelvecchi, 2016). Considering the small number of animals, rs-OIS appears to be a highly reliable tool, sensitive enough to pick up differences in lesion load.

## Conclusions

An analysis of resting state networks in the developing rodent brain together with the impact of inflammatory white matter injury was presented using fc measurements and machine learning techniques. Our approach was based on creating group-level fc statistical maps where a significant decrease in connectivity was observed in the case of the injured group; furthermore, seed-based fc measures were analyzed through SVM to identify connectivity patterns differences between NaCl and LPS groups. Those same fc measures were fed to an ANN that allowed the prediction of fractional lesion volume. Different fc approaches (ICA, graph theory, clustering, etc.) and alternatives to the chosen machine learning algorithms need further investigation on the basis of a larger sample. Another drawback of this study is the assumption of stationarity in fc, thus requiring the consideration of dynamic models in future studies (Hansen et al., 2015). Nevertheless, the translation of both machine-learning approaches, to a bedside optical monitoring system, such as fNIRS, could be helpful in providing early diagnosis in preterm infants especially when evaluating small cohorts.

## Author contributions

EG, FL, and GL conceived and designed the experiments; WP, CT, LA, IL, and GL performed the experiments; EG, CT, LA, IL, and GL analyzed the data; FL and GL contributed materials and analysis tools; EG, CT, FL, and GL wrote the paper.

### Conflict of interest statement

The authors declare that the research was conducted in the absence of any commercial or financial relationships that could be construed as a potential conflict of interest. The reviewer RL and handling Editor declared their shared affiliation, and the handling Editor states that the process nevertheless met the standards of a fair and objective review.
